# Future direction of pathogenesis and treatment for rheumatic disorders

**DOI:** 10.1186/ar3556

**Published:** 2012-02-09

**Authors:** Steffen Gay

**Affiliations:** 1 Department of Rheumatology, University Hospital, Gloriastrasse 25, CH-8091 Zurich, Switzerland

## 

After the breakthrough in the treatment of rheumatoid arthritis and numerous related disorders with biological therapies targeting TNFa at the Kennedy Institute in London

Millions of patients have tremendously benefitted. However, we cannot cure these diseases yet and have to search for additional therapeutic targets.

Since it was shown that synovial fibroblasts (SF) are not only effector cells responding to inflammatory stimuli, but appear endogenously activated and potentially involved into spreading the disease [1], we searched for the epigenetic modifications leading to the activated phenotype of these cells.

Epigenetics in its scientific definition "is the study of all heritable and potentially reversible changes in genome function that do not alter the nucleotide sequence within the DNA", but might be considered in simpler terms as the *regulation of gene expression*.

Epigenetic modifications include:

Acetylation,

Methylation,

Phosphorylation,

Sumoylation,

miRs or microRNAs.

Our laboratory is studying these processes and we have found that RASF reside in a hyperacetylated synovial tissue and appear hypomethylated [2]. Hypomethylation leads to the activated phenotype of RASF which is characterized by the production of matrix-degrading enzymes and of potent chemokines induced by Toll-like receptor signalling. Current strategies are designed to methylate these cells to deactivate and "normalise" them again.

miRs are about 20 nucleotide long smallRNAs acting to destroy specific mRNA.

In the race to identify specific miRs as novel targets we have identified for example, that interleukin-6 modulates the expression of the Bone Morphogenic Protein Receptor Type II through a novel STAT3microRNA cluster 17/92 pathway, which helps to explain the loss of the BMPR2 in the vascular cells in pulmonary hypertension [3]. Moreover, miR-203 is regulating the production of IL-6 [4].

Most interestingly, epigenetic therapy is also on the horizon [5].

## References

[B1] Lefèvre S Synovial fibroblasts spread rheumatoid arthritis to unaffected joints Nat Med 2009 15 1414 20 10.1038/nm.2050 19898488 PMC3678354

[B2] Karouzakis E Epigenetic control in rheumatoid arthritis synovial fibroblasts Nat Rev Rheumatol 2009 5 266 72 10.1038/nrrheum.2009.55 19412193

[B3] Brock M Interleukin-6 modulates the expression of the bone morphogenic protein receptor type II through a novel STAT3-microRNA cluster 17/92 pathway Circ Res 2009 104 1184 91 10.1161/CIRCRESAHA.109.197491 19390056

[B4] Stanczyk J Altered expression of miRNA-203 in rheumatoid arthritis synovial fibroblasts and its role in fibroblast activation Arthritis Rheum 2011 63 373 81 10.1002/art.30115 21279994 PMC3116142

[B5] Willyard C The saving switch Nat Med 2010 16 16 8 10.1038/nm0110-16 20057412

